# Click-crosslinkable and photodegradable gelatin hydrogels for cytocompatible optical cell manipulation in natural environment

**DOI:** 10.1038/srep15060

**Published:** 2015-10-09

**Authors:** Masato Tamura, Fumiki Yanagawa, Shinji Sugiura, Toshiyuki Takagi, Kimio Sumaru, Toshiyuki Kanamori

**Affiliations:** 1 National Institute of Advanced Industrial Science and Technology (AIST), Department of Life Science and Biotechnology, Tsukuba, 605-8565, Japan

## Abstract

This paper describes the generation of “click-crosslinkable“ and “photodegaradable“ gelatin hydrogels from the reaction between dibenzocycloctyl-terminated photoclevable tetra-arm polyethylene glycol and azide-modified gelatin. The hydrogels were formed in 30 min through the click-crosslinking reaction. The micropatterned features in the hydrogels were created by micropatterned light irradiation; the minimum resolution of micropatterning was 10-*μ*m widths for line patterns and 20-*μ*m diameters for circle patterns. Cells were successfully encapsulated in the hydrogels without any loss of viability across a wide concentration range of crosslinker. In contrast, an activated-ester-type photocleavable crosslinker, which we previously used to prepare photodegradable gelatin hydrogels, induced a decrease in cell viability at crosslinker concentrations greater than 1.8 mM. We also observed morphology alteration and better growth of cancer cells in the click-crosslinked photodegradable gelatin hydrogels that included matrigel than in the absence of matrigel. We also demonstrated micropatterning of the hydrogels encapsulating cells and optical cell separation. Both of the cells that remained in the non-irradiated area and the cells collected from the irradiated area maintained their viability.

Hydrogels composed of natural polymers (e.g., collagen, gelatin, and matrigel) have excellent biological functions that help to maintain cell viability and can enhance specific cellular functions^1^. For example, hepatocytes sandwiched between collagen hydrogels have been shown to exhibit a higher secretion rate of albumin than those in monolayer culture^2^. Gelatin is denatured collagen that retains cell binding sites.; therefore, it has been widely used to coat cell culture plates and to construct scaffolds for tissue engineering^3^. In addition, engineered gelatins, such as photocrosslinkable gelatin, have also been used for microscale tissue engineering because of their biocompatibility, biodegradability, and cell-binding capacity^4^,^5^,^6^. As another example, matrigel has been used as a three-dimensional culture model for cancer cells. Cancer cells in matrigel exhibit morphology alteration depending on the malignancy^7^,^8^, and this culture model should be used to distinguish normal cells from malignant bronchial epithelial cells^9^. Matrigel contains extracellular matrices (ECMs) composed of approximately 8% entactin, 30% collagen IV, and 60% laminin. Matrigel also contains growth factors, such as tumor growth factor-platelet-derived growth factor, and epidermal growth factor according to its manufacturers (Corning, Tewksbury, MA). The growth factors in matrigel induce remodeling of the cytoskeleton, modulation of cell attachment, and cell migration through the activation of a G-protein^10^,^11^, which alter the cell’s morphology through the involvement of actin dynamics^12^.

Light irradiation is a useful technique to control the spatiotemporal cell culture microenvironment^13^ and to stimulate cells^14^,^15^ because light can act in a microscale and noncontact manner. Anseth *et al.* developed photodegradable hydrogels as a powerful tool to regulate the microenvironment of hydrogels^16^. They fabricated cylindrical wells as small as 5 *μ*m in diameter in the hydrogels, and also synthesized photodegradable hydrogels by using a click reaction between polyethylene glycol (PEG)-tetra difluorinated cyclooctyne (DIFO3) and bis(azide)-functionalized polypeptides^17^. They also found that human mesenchymal stem cells could be encapsulated in these click hydrogels while maintaining cell viability, and that encapsulated 3T3 fibroblasts grew in the degraded hydrogel in line with the pattern of photodegradation.

PEG-based hydrogels have been widely used for tissue engineering; however, they are generally biologically inert. Although modification with RGD peptide can be effective in some cases as shown in the above mentioned click closslinkable photodegradable hydrogels^17^, the specific mechanism behind the interaction between the cells and the scaffold is often unknown. Therefore, hydrogels composed of natural polymers are desirable for certain specific applications because natural polymers have excellent biological functions as described above. To overcome this problem, we previously developed photodegradable gelatin hydrogels by use of crosslinking with *N*-hydroxysuccinimide (NHS)-terminated photocleavable tetra-arm PEG, which is composed of amine-reactive NHS-activated ester groups and tetra-arm PEG with photocleavable *o*-nitrobenzyl groups (NHS-PC-4armPEG)^18^. We performed micropatterned degradation of these hydrogels and estimated the minimum resolution of degradation to be 20 *μ*m on the basis of circles formed in the hydrogels. We also applied these photodegradable gelatin hydrogels to optical cell separation, namely, cells encapsulated in the photodegradable hydrogel could be separated by local photodegradation^19^. Although we were able to demonstrate optical cell separation without cell death, we encountered a problem in applying this technique to morphology-based cancer cell separation. NHS-activated ester group theoretically react with any amine group in the pregel solution; therefore, it is difficult to include additional components such as growth factors in the hydrogel. In addition, NHS-activated ester groups react with proteins on cell surfaces, and this reaction would probably disturb the morphology alteration of the cancer cells in the hydrogels. To overcome these issues, we use a click reaction to prepare photodegradable gelatin hydrogels. The click reaction is a convenient reaction for synthesizing biomaterials because it proceeds in aqueous solution without involving any other biological compounds, such as amino acids, proteins, lipids, or hydrocarbons^20^. Click reactions can, therefore, avoid the loss of the biological functions of these molecules^21^. The first click reactions required copper as a catalyst^22^; however, these reactions can now be done under copper-free conditions by utilizing a strained cyclooctyne structure^23^,^24^. The copper-free click reactions can be used for various biological studies including cell culture studies^25^,^26^. Examples of copper-free click reactions include (i) strain-promoted azide-alkyne cycloaddition (SPAAC), (ii) thiol-ene photocoupling, (iii) the Diels-Alder reaction, (iv) the inverse electron demand Diels-Alder reaction, (v) the tetrazole-alkene photo-click reaction, and (vi) the oxime reaction^26^. In these copper-free click reactions, SPAAC forms a covalent bond within approximate one hour of mixing compounds containing alkyne and azide groups.

In this study, we hypothesized that a copper-free click reaction, SPAAC, would be more biocompatible than an NHS activated-ester reaction for encapsulating cells in gelatin-based photodegradable hydrogels. We synthesized a photodegradable gelatin (PD-gelatin) hydrogels by using a click reaction between azide-modified gelatin and dibenzocycloctyl-terminated photoclevable tetra arm-PEG (DBCO-PC-4armPEG). We characterized the physicochemical properties of DBCO-PC-4armPEG and the photodegradation properties of the PD-gelatin hydrogels. We evaluated the cell compatibility, including cell viability and cellular morphology, of the PD-gelatin hydrogels synthesized by using the click reaction and compared it with that of hydrogels synthesized by using the NHS activated-ester reaction. We also demonstrated cell micropatterning and optical cell separation in the PD-gelatin hydrogels by using micropatterned light irradiation.

## Results

### Characterization of azide-modified gelatin and DBCO-PC-4armPEG

**Figure 1:**
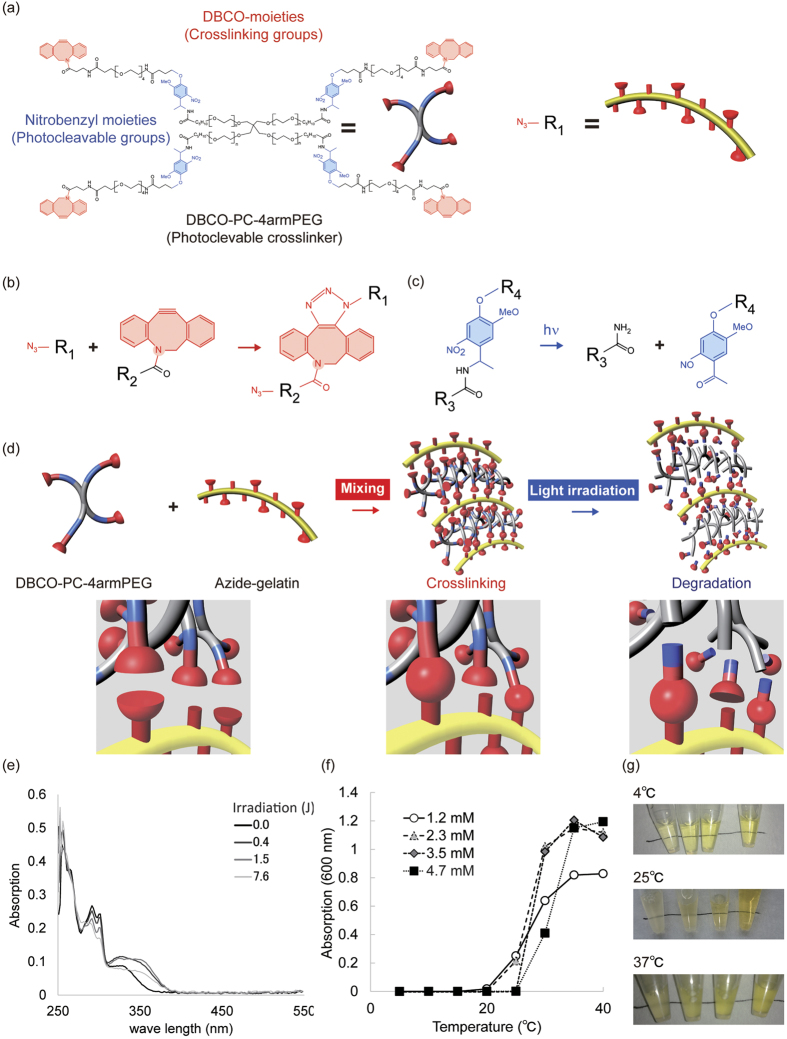
Schematic diagram of click-crosslining hydrogel formation and photodegradation by light irradiation. (**a**) Molecular structure of DBCO-PC-4armPEG and azide-modified gelatin. R_2_ indicates gelatin. (**b**) Crosslinking reaction between DBCO-PC-4armPEG and azide-modified gelatin to form photodegradable hydrogels. (**c**) Photocleavage reaction induced by light irradiation. (**d**) Schematic representation for the formation and degradation of photodegradable hydrogels. (**e**) Change in the absorption spectra induced by light irradiation. (**f**) Temperature-dependent turbidity changes in aqueous solutions of DBCO-PC-4armPEG. (**g**) Photographs showing the visible color and transparency of the aqueous solutions of DBCO-PC-4armPEG. The concentration of DBCO-PC-4armPEG was 1.2, 2.3, 3.5, and 4.7 mM from left to right. The scheme and images in ((**a)** to (**d**)) was drawn by M.T., F. Y. and S.S. The photographs in (**g**) were taken by M.T.

The modification rate of the azide-modified gelatin was estimated as 37, 67, 87, and 98 mol% for azide-gelatin (25), azide-gelatin (50), azide-gelatin (75), and azide-gelatin (100), respectively (Table 1). Interestingly, the aqueous solution of the azide-modified gelatin (25 mg/mL) did not form a hydrogel at 25 °C, even though an aqueous solution of natural gelatin formed a hydrogel under the same concentration and temperature conditions (Table S2, supporting information). This phase behavior is convenient for the preparation of PD-gelatin hydrogels because the pregel polymer solutions can be handled at room temperature. We next synthesized the click-crosslinkable and photocleavable crosslinker DBCO-PC-4armPEG. The main structure of DBCO-PC-4armPEG is a tetra-arm polyethylene glycol that terminates with *o*-nitrobenzyl and DBCO groups (Fig. 1a). DBCO groups form covalent bonds via the click reaction with azide groups (Fig. 1b), and o-nitrobenzyl groups are cleaved upon light irradiation (Fig. 1c). Through this molecular mechanism, DBCO-PC-4armPEG and azide-modified gelatin form a hydrogel, via the click reaction, that can be degraded by light irradiation (Fig. 1d). To evaluate photocleavage of DBCO-PC-4armPEG, we measured the absorption spectra of DBCO-PC-4armPEG solutions before and after light irradiation. The absorption peaks at 292 and 310 nm decreased depending on the light irradiation dose, whereas the absorption between 350 to 400 nm increased depending on the light irradiation dose (Fig. 1e). This result indicates that the *o*-nitrobenzyl groups in DBCO-PC-4armPEG were photocleaved, as we previously reported in our study on NHS-PC-4armPEG^18^. The turbidity of the DBCO-PC-4armPEG solution increased at temperatures over 30 °C (Fig. 1f,g). On the basis of this result, we prepared PD-gelatin hydrogels at room temperature for our subsequent studies.

### Formation and degradation of PD-gelatin hydrogels

**Figure 2:**
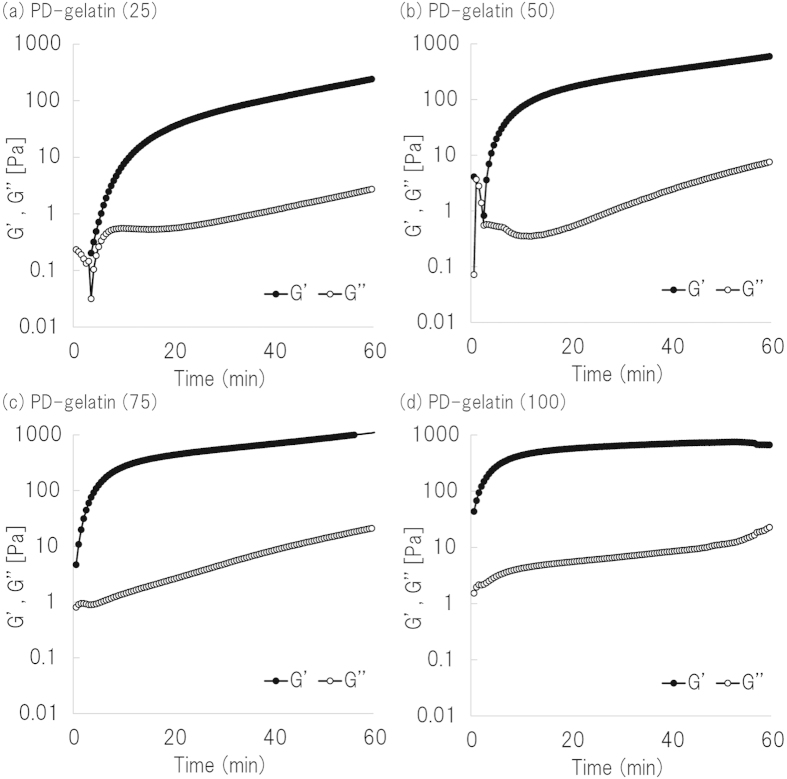
Time sweep profiles of the elastic modulus (G’) and viscous modulus (G”). The composition of the pregel solution was the same as that shown in Table S1 for PD-gelatin (25) (**a**), PD-gelatin (50) (**b**), PD-gelatin (75) (**c**) and PD-gelatin (100) (**d**).

**Figure 3:**
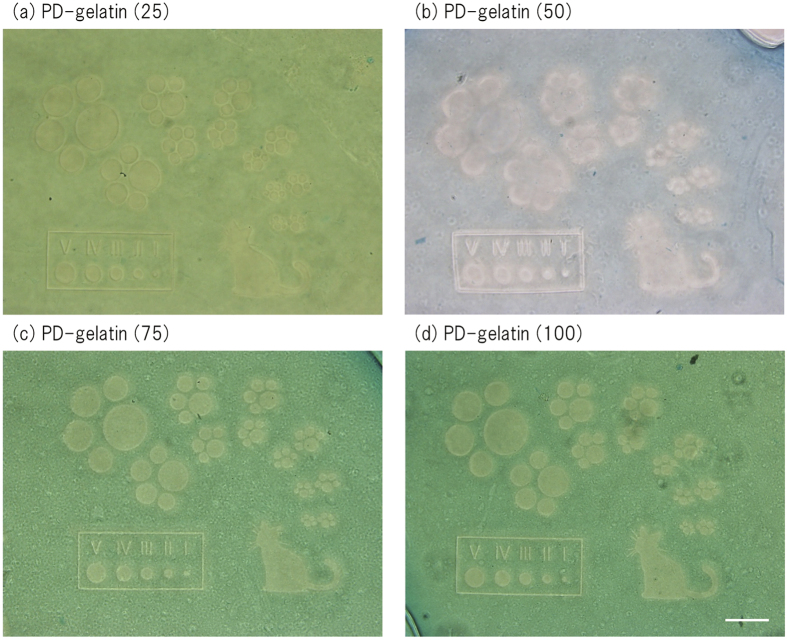
Micropatterning of PD-gelatin hydrogels by using a PC-controlled microprojection system. PD-gelatin hydrogels of PD-gelatin (25) (**a**), PD-gelatin (50) (**b**), PD-gelatin (75) (**c**), and PD-gelatin (100) (**d**) were exposed to micropatterned light for 30 s. The image used for micropatterning is shown in Figure S4. The diameters of the circles in the image were approximately 20 (I), 30 (II), 45 (III), 68 (IV), and 101 *μ*m (V). The line width of the frame border was approximately 10 *μ*m. Scale bars = 200 *μ*m.

PD-gelatin hydrogels were formed by using the azide-modified gelatin with different modification rates. We measured the the elastic and viscous moduli with time to determine the gelation time (Fig. 2). For all four types of aside-modified gelatin, the value of G’ quickly increased within 5 min and continuously increased for 50 min depending on the azide modification rate. Taking into account this rheology measurement and the desirable time for cell encapsulation, we set the incubation time for gelation as 30 min for our subsequent experiments. We also characterized micropatterned degradation of PD-gelatin hydrogels. Figure S4 shows the image used for micropatterning, and Fig. 3 shows the created micropattern in the PD-gelatin hydrogels. The minimum resolution of micropatterned degradation was 10 *μ*m in width for the line patterns and 20 *μ*m in diameter for the circle patterns. The created micropattern in the hydrogels appeared to be sharp with increasing concentrations of DBCO-PC-4armPEG, indicating that the crosslinking density plays an important role in creating and maintaining the small structure.

### Cell viability in PD-gelatin hydrogel

**Figure 4:**
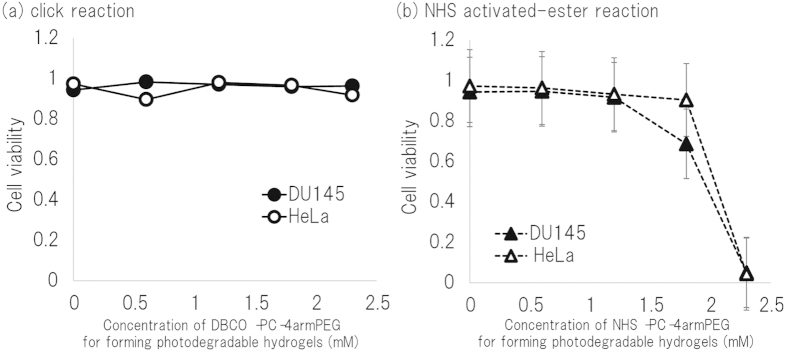
Viability of HeLa and DU145 cells encapsulated in PD-gelatin hydrogel prepared by using a click reaction (a) or an NHS activated-ester reaction (b). The hydrogels used in this study were composed of PD-gelatin (25), PD-gelatin (50), PD-gelatin (75), and PD-gelatin (100).

To confirm the biocompatibility of our PD-gelatin hydrogel, we encapsulated HeLa and DU145 cells in the PD-gelatin hydrogels. Cell viability in the PD-gelatin hydrogels was measured by using the LIVE/DEAD assay and was compared with that in hydrogels prepared by using the NHS activated-ester reaction and NHS-PC-4armPEG, which we previously studied^18^. There was no detectable cell death among either the HeLa cells or the DU145 cells in the PD-gelatin hydrogels prepared by using the click reaction (Fig. 4a). In contrast, cell death was observed among cells in the PD-gelatin hydrogel that was prepared by using the NHS activated-ester reaction when the concentration of the crosslinker exceeded 1.7 mM (Fig. 4b).

### Matrigel-containing PD-gelatin hydrogels

**Figure 5:**
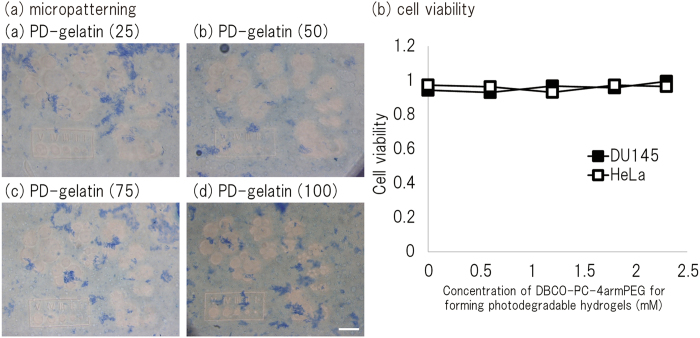
Micropatterning (a) and cell viability (b) in PD-gelatin hydrogels containing matrigel. The hydrogels used in this study were composed of PD-gelatin (25)_M+, PD-gelatin (50)_M+, PD-gelatin (75)_M+, or PD-gelatin (100)_M+ as shown in Table S1. The hydrogels were exposed to micropatterned light (365 nm, 156 mW/cm^2^) for 30 s. The image used for micropatterning is shown in Figure S4. The diameters of the circles in the image were approximately 20 (I), 30 (II), 45 (III), 68 (IV), and 101 *μ*m (V). The line width of the frame border was approximately 10 *μ*m. Scale bars = 200 *μ*m. The micropattern was formed by light irradiation using photo-mask free apparatus. Scale bar = 200 *μ*m.

**Figure 6:**
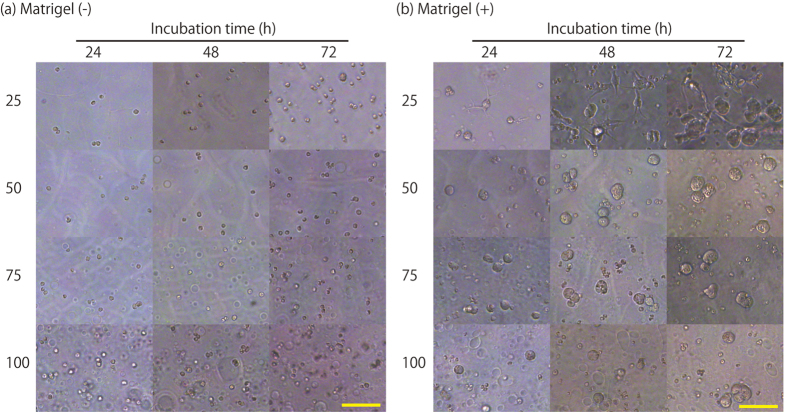
Microscopic phase contrast images of HeLa cells encapsulated in the PD-gelatin hydrogel without matrigel (a) and with matrigel (b). The numbers (25, 50, 75, and 100) to the left of the images indicate the composition of the hydrogels, PD-gelatin (25), PD-gelatin (50), PD-gelatin (75), and PD-gelatin (100) shown in Table S1. Scale bars = 100 *μ*m.

Generally, click reactions do not induce reactions with other biological compounds. This characteristic of click reactions can be an advantage for encapsulating cells in hydrogels because some biological compounds are required for particular cell culture applications. To confirm the formation of PD-gelatin hydrogels containing other biological compounds, we prepared PD-gelatin hydrogels containing matrigel (Table S1), which contains various biological compounds that affect cellular functions. We created a micropattern in the PD-gelatin hydrogels containing matrigel (Table S1) and observed a blue-colored aggregate after CBB staining (Fig. 5a). This aggregate was probably formed when we mixed the azide-gelatin with the matrigel at 25 °C because the polymer content in matrigel forms molecular aggregates at room temperature even at the low concentration used in this study (0.5 mg/mL). We did not detect any death among either the HeLa cells or the DU145 cells in the PD-gelatin hydrogels containing the matrigel (Fig. 5b). In fact, the HeLa cells in the PD-gelatin hydrogels with matrigel exhibited considerable cell growth and formed a colony as large as 50 *μ*m in 3 days of culture (Fig. 6). Notably, some of the HeLa cells in the PD-gelatin (25) hydrogel with matrigel formed invadopodia, which has not been observed in other hydrogels (Fig. 6b). In contrast, the morphology of the HeLa cells in the PD-gelatin hydrogels without matrigel showed no notable change (Fig. 6a). These results indicate that the matrigel contents (e.g. growth factors) supported cellular invasion of HeLa cells in the PD-gelatin hydrogel. PD-gelatin hydrogel containing matrigel could not be prepared using the NHS activated-ester-type crosslinker under the conditions with the same concentrations of gelatin and crosslinker shown in Table S1, probably because the NHS activated-ester groups reacted with the amino groups of the biological compounds in the matrigel.

### Cell cultures, micropatterning, and optical cell separation in PD-gelatin hydrogels

**Figure 7:**
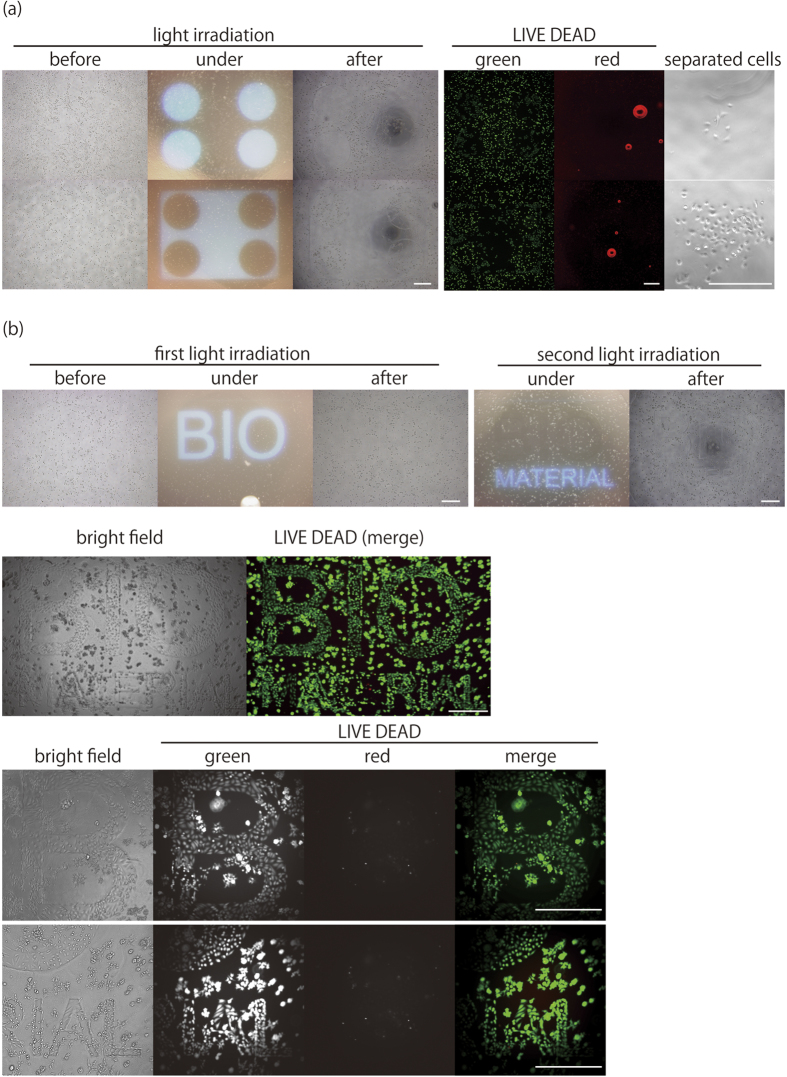
Cell micropatterning and optical cell separation using PD-gelatin hydrogels. HeLa cells were encapsulated in PD-gelatin (25)_M+ hydrogels and cultured for 24 h. (**a**) Micropattering of hydrogels and optical cell separation. The hydrogel contaning cells was exposed to four circles of micropatterned light (a, left). Fluorescent images were obtained after LIVE/DEAD staining (a, middle). Cells in the degraded area were collected, transfered into another culture dish, and incubated for 24 h (a, right). (**b**) Stepwise cell micropatterning by repeating micropatterned light irradiation. The first pattern of “BIO” was irradiated to the hydrogel (b, top left) and incubated for 24 h, then the second pattern of “MATERIAL” was irradiated to the hydrogel (b, top right) and incubated for another 48 h to assess cell growth. Fluorescent images (b, middle and bottom) were obtained after LIVE/DEAD staining. Scale bars = 200 *μ*m

Finally, we demonstrated cell micropatterning and optical cell separation using PD-gelatin hydrogels (Fig. 7). HeLa cells were encapsulated in the PD-gelatin (25)_M+ hydrogels containing matrigel. The cells in the irradiated area were successfully removed after micropatterned light irradiation (Fig. 7a, left). The cells were collected from the irradiated area by pipetting in another culture dish. The remaining cells in the non-irradiated area were still viable after 24 h (Fig. 7a, middle). The separated cells that were transferred to the culture dish regrew in the dish (Fig. 7a, right). We also demonstrated stepwise cell micropatterning using the PD-gelatin hydrogel. First, the micropatterned light showing the word “BIO” was irradiated to the hydrogel (Fig. 7b, top left). Then, after 24 h, another micropatterned light showing the word “MATERIAL” was irradiated to the hydrogel (Fig. 7b, top right). After 72 h, the cells remaining in the irradiated area grew two-dimensionally, indicating that the hydrogel was degraded and that the cells were growing on the surface of the dish (Fig. 7b, middle and bottom). After LIVE/DEAD staining, the green colored word “BIO MATERIAL” appeared as a result of the cells growing in the degraded area.

## Discussion

In this study, we prepared PD-gelatin hydrogels via the reaction between azide-modified gelatin and DBCO-PC-4armPEG. In our preliminary experiment, we synthesized azide-PC-4armPEG and DBCO-gelatin by using another approach. However, the DBCO-gelatin formed an insoluble aggregate in aqueous buffer depending on the modification rate of the DBCO (Figure S5, supporting information). We therefore synthesized DBCO-PC-4armPEG and azide-modified gelatin in this study. Azide-modification of the gelatin improved the usability of the gelatin solution to prepare the PD-gelatin hydrogels. The aqueous solution of azide-modified gelatin (25 mg/mL) maintained its liquid state at 25 °C (Table S2, supporting information), which enabled us to handle the pregel solutions at room temperature. The minimum resolution of micropatterning in the PD-gelatin hydrogel was 10 to 20 *μ*m, which is similar in size to cells, meaning that our technique was capable of micropatterning with sufficiently high resolution to be applied to optical cell separation. In the experiments with PD-gelatin hydrogel containing matrigel, aggregates of matrigel were found in the hydrogel after CBB staining (Fig. 5), yet cell growth in the hydrogel with matrigel was clearly improved relative to that without matrigel (Fig. 6). The aggregates likely comprised matrix polymers, such as collagen and laminin, because matrix polymers form the aggregates at 37 °C. Factors such as EGF, PDGF, and VEGF appeared to have been uniformly mixed in the pregel solution because there was no significant difference in cell growth across the entire surface of the culture dish (data not shown). Compared to our previous study in which we used the NHS activated-ester reaction to prepare photodegradable hydrogels, the click reaction provided several advantages: (i) the click reaction did not cause any detectable cytotoxicity over a wide concentration range, whereas the NHS-activated ester reaction induced cell damage at crosslinker concentrations greater than 1.8 mM (Figs 4 and 5); (ii) the click reaction enabled us to prepare PD-gelatin hydrogels that contained additional biological compounds (e.g., matrigel) to stimulate cells (Figs 5 and 6). These advantages broaden the potential applications of the cell culture platform of photodegradable hydrogels. The ability to prepare hydrogels with a wide range of crosslinker concentrations allows the encapsulation of cells in stiffer hydrogels. Since stiffness is an important microenvironmental factor to control cell behavior^13^,^27^, the potential to vary stiffness is important for the application of PD-gelatin hydrogels to tissue engineering. Clearly, the ability to prepare hydrogels containing a functional biological compound is also important for the application of PD-gelatin hydrogels to tissue engineering.

There has been pioneering work to prepare photodegradable hydrogels by using click reactions^28^. Building on this previous work, here, we demonstrated that photodegradable hydrogels can be prepared using a natural polymer, gelatin, in the click reaction. Recently, PEG-based synthetic hydrogels have been recognized as potential materials for cancer research through the introduction of cell binding and degradation motifs^29^. Yet, the specific molecular mechanisms behind the interactions between particular cells and the matrix are usually unknown, as exemplified by the fact that matrigel, which is a mixture of various ECMs and growth factors, is currently widely used as a versatile scaffold. In this context, the use of a natural polymer for the preparation of photodegradable hydrogels offers considerable versatility and a realistic approach depending on the application. For example, we observed significant cell growth and morphology alteration in PD-gelatin hydrogels containing matrigel. This property is beneficial for the application of hydrogels to optical separation of cancer cells combined with morphological analysis^30^. In contrast, the growth of cells in PEG-based photodegradable hydrogels has been shown to be very slow; mesenchymal stem cells formed a colony smaller than 50 *μ*m after 20 days of culture^16^, and 3T3 cells grew only in the degraded area^10^. In addition, hydrogels prepared with gelatin derivatives should be biodegradable as reported for photocrosslinkable gelatin^4^. This biodegradable property of gelatin-based hydrogel represents another advantage over PEG-based synthetic hydrogels. We demonstrated micropatterning of the hydrogels encapsulating cells and optical cell separation (Fig. 7). The cells that remained in the non-irradiated area and the cells collected from the irradiated area maintained their viability. Of particular note, the cells collected from the degraded area attached to the surface of the dish and grew (Fig. 7a). These results indicate that the optical cell separation process from the PD-gelatin hydrogels caused negligible cytotoxicity. Stepwise cell micropatterning showed that the created micropattern was maintained for at least 96 h, and that encapsulated cells grew well both in the PD-gelatin hydrogel and in the degraded area with high viability (Fig. 7b). These results indicate that PD-gelatin hydrogel could be used as a photocrosslinkable gelatin scaffold for microscale tissue engineering^4^,^13^. For example, we are currently working on the application of PD-gelatin hydrogels to 3D cell micropatterning and perfusable tissue engineering. In the pilot study on 3D cell micropatterning, we observed the different cell growth in the degraded area compared to the non-degraded area (data not shown). In another pilot study, we could form the co-culture of two-different cells in the degraded microchannel structure and non-degraded area (data not shown). We will report the details of these applications in the near future.

## Methods

### Materials

Azide-PEG_4_-NHS ester (Click Chemistry Tools LLC. Scottsdale, AZ), DBCO-PEG_4_-amine (Click Chemistry Tools, LLC.), 4-(2-hydroxyethyl)-1-piperazineethanesulfonic acid (HEPES, Wako Pure Chemical Industries, Ltd., Osaka, Japan), and Corning matrigel matrix (Corning) were purchased and used without further purification. All other reagents were purchased from Sigma-Aldrich Co. LLC. (St. Louis, MO) unless specified. HEPES buffer (300 mM, pH 7.4) was filtrated through 0.2 *μ*m pore filter (Millipore Co., Billerica, MA) before use.

### Synthesis of azide-modified gelatin

Four types of azide-modified gelatin were synthesized by mixing gelatin with Azide-PEG_4_-NHS ester solutions (Figure S1, supporting information) at the mixing ratio shown in Table 1. The mixing ratio was designed on the basis of the capacity for azide modification, which was estimated from the amount of primary amino groups in gelatin^31^. A gelatin solution in HEPES buffer (2.5 mL) and an azide-PEG_4_-NHS ester solution in 10 mM phthalate acid buffer (pH 4.0) (2.5 mL) were prepared in separate tubes. Both solutions were then mixed in a tube and incubated at 37 °C for 2 h for conjugation. This reaction mixture was then transferred into a dialysis tube (MWCO 6–8 k, Spectrum laboratories, Inc., Rancho Domingues, CA) and dialyzed against 5 L of MilliQ water (Millipore, Billerica, USA) for 24 h; the MilliQ water was changed at 30 min, 1, 3, 5, and 7 h after dialysis was initiated. The dialyzed reaction mixture was freeze-dried (FDS-1000, Tokyo Rikakikai Co., Ltd., Tokyo, Japan), and azide-modified gelatin was obtained with a yield between 71% and 82%. The obtained azide-modified gelatin was dissolved in HEPES buffer at 37 °C, and the solution was stored at 4 °C. The modification rate of the azide-modified gelatin was calculated from the amount of remaining reactive amino groups, measured by a fluorescamine assay, in comparison to that in original gelatin^32^. Briefly, gelatin and azide-modified gelatin were diluted to 1.25 mg/mL in HEPES buffer. The solution (90 *μ*L) was added to a 3 mg/mL fluorescamine solution in dimethylsulfoxide (DMSO) (30 *μ*L). The mixture was then incubated for 30 min at room temperatureunder shielding from light, and the fluorescence intensity was measured by plate reader at ex. 360 nm and em. 465 nm (GENios, Tecan Japan Co., Ltd, Kanagawa, Japan).

### Synthesis of DBCO-PC-4armPEG

NHS-PC-4armPEG was synthesized according to our previous report^18^. DBCO-PC-4armPEG was synthesized by the reaction between NHS-PC-4armPEG and DBCO-PEG_4_-amine (Figure S2, supporting information). DBCO-PEG_4_-amine (600 mg) was added to NHS-PC-4armPEG (3.018 g) in 125 mL of DMSO, and then incubated for 40 °C while stirring. The reaction product was purified by the following procedure. The solution was dripped into 1 L of diethylether and cooled on ice for 30 min. After the supernatant was removed by decantation, the precipitate was dissolved in 10 mL of tetrahydrofuran (THF). Subcequently, the THF solution was dripped into 400 mL of diethylether and the precipitate was obtained. This precipitation process was repeated three times. After precipitation, 3.4 g of DBCO-PC-4armPEG was obtained (yield: ca. 99%) (Mw 10,998). The structure was determined by means of ^1^H-NMR in deuterated chloroform. The NMR spectra of the DBCO-PC-4armPEG showed peaks from the aromatic ring in the DBCO group at approximately 7.3–7.6 ppm and a peak from the methylene adjoining the amino group in DBCO at 5.1 ppm (Figure S3a, supporting information). Compared to the NHS-PC-4armPEG, the NMR spectra of the DBCO-PC-4armPEG showed the loss of a peak from an NHS activated ester group at 2.9 ppm, and the appearance of peaks at 7.3–7.6 and 5.1 ppm (Figure S3b, supporting information).

### Absorption spectra measurement of DBCO-PC-4armPEG cleaved by light irradiation

The photoinduced cleavage of DBCO-PC-4armPEG was detected by changes in its absorption spectra. A 30-*μ*L drop of the DBCO-PC-4armPEG solution (120 *μ*M) in HEPES buffer in a micro tube was irradiated with light from a UV light source (365 nm, 25.2 mW/cm^2^: UVE-251S, San-Ei Electric Co. Ltd., Osaka, Japan), and the absorption spectra from 300 to 600 nm were measured at 10 °C (Nanodrop, Thermo Fisher Scientific K.K., Kanagawa, Japan).

### Turbidity measurement of DBCO-PC-4armPEG at different temperatures

Solutions of DBCO-PC-4armPEG in HEPES buffer were prepared at concentrations of 1.2, 2.3, 3.5, and 4.7 mM. For turbidity measurement, absorbance at 600 nm was measured in the temperature range of 5 to 40 °C by using a spectrophotometer (V-560, JASCO Corporation, Tokyo, Japan) with a temperature controller (ETC-505T, JASCO); the temperature was increased stepwise (5 °C every 100 sec).

### Preparation of PD-gelatin hydrogels via a click reaction

The azide-modified gelatin solution and DBCO-PC-4armPEG solution were prepared in HEPES buffer. Hydrogel formation by the click reaction was performed by mixing equal volumes of these solutions at the concentration shown in Table S1 (supporting information). Ten microliters of the mixture was immediately pippeded on the gap between a Teflon rod with spacers and a poly-L-lysine (PLL) coat 35 mm dish (Corning) to form a 300 *μ*m thick PD-gelatin hydrogel, which was then incubated at room temperature for 30 min. The PD-gelatin hydrogel containing matrigel was prepared similarly by using an azide-modified gelatin solution containing 1 mg/mL of protein in matrigel. Note that matrigel typically contains 9–12 mg/mL protein including ECMs and growth factors according to the information provided by the company (Corning).

### Rheology measurement of PD-gelatin hydrogels

The time required for gelation was estimated by measuring the elastic modulus and the viscous modulus (G’ and G”) by a MCR-302 rheometer (Anton Paar Ltd., Graz, Austria). PD-gelatin hydrogels were prepared by the method outlined in above section. Immediately after mixing the azide-modified gelatin and the DBCO-PC-4armPEG solutions, 500 *μ*L of the PD-gelatin hydrogels was on the plate in a rheomete. G’ and G” values were continuously monitored at 25 °C for 50 min.

### Micropatterned photodegradation in the hydrogel

Micropatterned light irradiation was performed by a PC-controlled microprojection DESM-01 system (Engineering System, Co. Ltd., Matsumoto, Japan)^33^,^34^. The micropattern was illustrated by using Adobe illustrator CS4 (Adobe systems software Ireland Ltd.). The PD-gelatin hydrogel was exposed to micropatterned light for 30 sec (365 nm, 156 mW/cm^2^), and incubated at 37 °C for 60 min. 0.01% Coomassie Brilliant Blue (CBB) (Dojindo, Kumamoto, Japan) was used to stain the micropatterned PD-gelatin hydrogel for 1 h, and then observed by a microscope (IX-71, Olympus Corporation, Tokyo, Japan).

### Viability study in PD-gelatin hydrogels

HeLa and DU145 cells were obtained from RIKEN Bioresource Center (Tsukuba, Japan). RPMI1640 (Life Technologies, Carlsbad, USA) supplemented with 10% inactivated fetal bovine serum (FBS), in a 5% CO_2_ at 37 °C. HeLa and DU145 cells were trypsinized, and resuspended by the azide-modified gelatin solution at 1.4 × 10^5^ cells/mL. Seven microliters of the azide-modified gelatin solution containing cells was mixed with 7 *μ*L of DBCO-PC-4armPEG solution, placed in a 300-*μ*m gap between a Teflon rod and a PLL coat 48-well plate, and incubated at room temperature for 30 min. After incubation, 500 *μ*L of the culture medium were gently added and the dish was incubated at 37 °C for 24 h. This solution for the LIVE/DEAD cell viability assay was prepared by mixing 1 *μ*L of calcein AM for live cells and 4 *μ*L of ethidium homodimer-1 for dead cells in 5 mL of PBS according to the information provided by the company (Life Technologies). The hydrogels were washed with PBS, and then incubated at 37 °C for 30 min in 200 *μ*L of LIVE/DEAD solution, and then images were captured by using a confocal microscope (FV300, Olympus). Live and dead cells were counted for more than 100 cells per sample.

### Cell cultures, micropatterning, and optical cell separation in PD-gelatin hydrogels

HeLa cells were trypsinized, and resuspended by the azide-modified gelatin solution at a concentration of 3.3 × 10^3^ cells/mL. PD-gelatin hydrogels were formed according to the above protocol for hydrogel preparation. Cell images were captured by a microscope (IX-71) after the cells were incubated for 24, 48, and 72 h. Micropatterning and optical cell separation was also performed by using the PC-controlled microprojection system according to the protocol outlined in section of “Micropatterned photodegradation in the hydrogel”.

## Additional Information

**How to cite this article**: Tamura, M. *et al.* Click-crosslinkable and photodegradable gelatin hydrogels for cytocompatible optical cell manipulation in natural environment. *Sci. Rep.* **5**, 15060; doi: 10.1038/srep15060 (2015).

## Supplementary Material

Supplementary Information

## Figures and Tables

**Table 1:** Preparation condition and analysis of azide-modified gelatin.

Type of azide-modified gelatin	Preparation condition			Modification rate (mol%)^b^
	Gelatin (mg/mL)	Azide-PEG_4_-NHS (mM)	Azide/-NH_2_ (mol%)^a^	
Gelatin		0	0	0 ± 1.5
Azide-gelatin (25)		4.7	25	37 ± 1.3
Azide-gelatin (50)	25	9.4	50	67 ± 0.5
Azide-gelatin (75)		14.1	75	87 ± 0.9
Azide-gelatin (100)		18.8	100	98 ± 0.2

^a^Mole ratio of azide to primary amino groups in gelatin was calculated from the amounts of amino group in gelatin^31^.

^b^Modification rate was calculated from the amount of reactive amino groups measured by fluorescamine assay before and after reaction.
