# Supplementary material for: Click-crosslinkable and photodegradable gelatin hydrogels for cytocompatible optical cell manipulation in natural environment
Source: Sci Rep. 2015 Oct 9;5:15060. doi: 10.1038/srep15060 (PMC4598855; doi:10.1038/srep15060)
Supplement: Supplementary Information [file srep15060-s1.doc]

**Supplementary Information**

Click-crosslinkable and photodegradable gelatin hydrogels for cytocompatible optical cell manipulation in natural environment

Masato Tamura, Fumiki Yanagawa, Shinji Sugiura*, Toshiyuki Takagi, Kimio Sumaru, and Toshiyuki Kanamori

Biotechnology Research Institute for Drug Discovery, National Institute of Advanced Industrial Science and Technology (AIST), Central 5th, 1-1-1 Higashi, Tsukuba, Ibaraki 305-8565, Japan

**Keywords**: Hydrogel, cell encapsulation, photodegradation, micropatterning, click chemistry

*Corresponding Author

Dr. Shinji Sugiura

E-mail: [shinji.sugiura@aist.go.jp](mailto:shinji.sugiura@aist.go.jp).

Phone: +81-29-861-6286

Fax: +81-29-861-6278

Biotechnology Research Institute for Drug Discovery, National Institute of Advanced Industrial Science and Technology (AIST), Central 5th, 1-1-1 Higashi, Tsukuba, Ibaraki 305-8565, Japan

**Table S1.** Preparation condition of photodegradable gelatin hydrogels

| Type of hydrogel | Type of azide-gelatin | Azide-gelatin (mg/mL) | DBCO-PC-4armPEG  (mM) | Matrigel  (mg/mL) |
| --- | --- | --- | --- | --- |
| Gelatin | Gelatin | 12.5 | 0 | 0 |
| PD-gelatin (25) | Azide-gelatin (25) | 12.5 | 0.6 | 0 |
| PD-gelatin (50) | Azide-gelatin (50) | 12.5 | 1.2 | 0 |
| PD-gelatin (75) | Azide-gelatin (75) | 12.5 | 1.8 | 0 |
| PD-gelatin (100) | Azide-gelatin (100) | 12.5 | 2.3 | 0 |
| Gelatin_M+ | Gelatin | 12.5 | 0 | 0.5 |
| PD-gelatin (25) _M+ | Azide-gelatin (25) | 12.5 | 0.6 | 0.5 |
| PD-gelatin (50) _M+ | Azide-gelatin (50) | 12.5 | 1.2 | 0.5 |
| PD-gelatin (75) _M+ | Azide-gelatin (75) | 12.5 | 1.8 | 0.5 |
| PD-gelatin (100) _M+ | Azide-gelatin (100) | 12.5 | 2.3 | 0.5 |

**Table S2.** Thermal sol-gel phase transition of azide-gelatin aqueous solution

| Type of azide-gelatin | Concentration (mg/mL) | Temperature  (°C) | Phase |
| --- | --- | --- | --- |
| Gelatin | 25 | 4 | Gel |
|  |  | 25 | Gel |
|  |  | 37 | Sol |
| Azide-gelatin (25) |  | 4 | Gel |
|  |  | 25 | Sol |
|  |  | 37 | Sol |
| Azide-gelatin (50) |  | 4 | Gel |
|  |  | 25 | Sol |
|  |  | 37 | Sol |
| Azide-gelatin (75) |  | 4 | Gel |
|  |  | 25 | Sol |
|  |  | 37 | Sol |
| Azide-gelatin (100) |  | 4 | Gel |
|  |  | 25 | Sol |
|  |  | 37 | Sol |


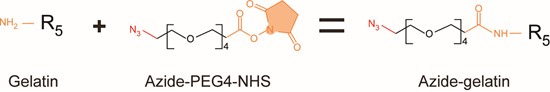


**Figure S1**. Schematic diagram of azide-modified gelatin formation by the NHS activated-ester reaction. R_5_ indicates gelatin. The scheme was drawn by M.T. and S.S.


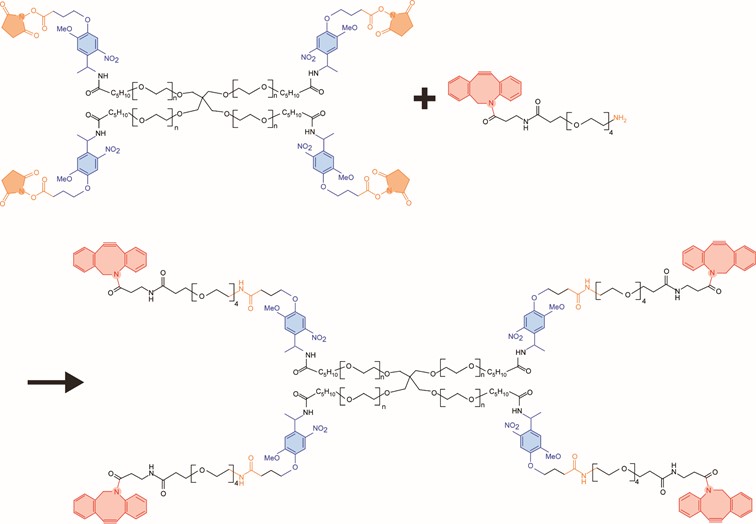


**Figure S2**. Schematic diagram of DBCO-PC-4armPEG formation by the NHS activated-ester reaction. The scheme was drawn by M.T and S.S.

(a)


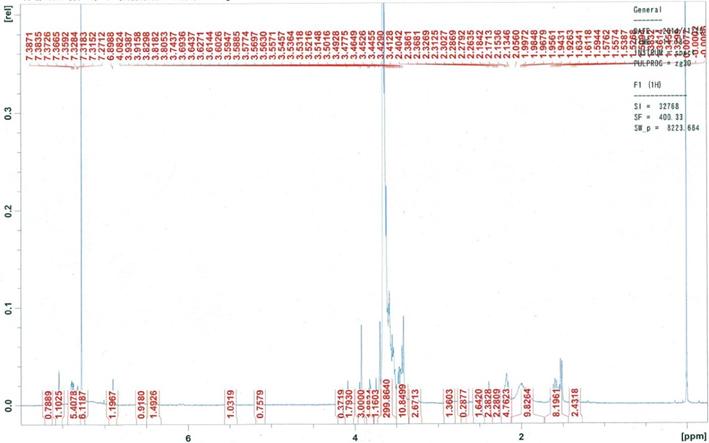


(b)


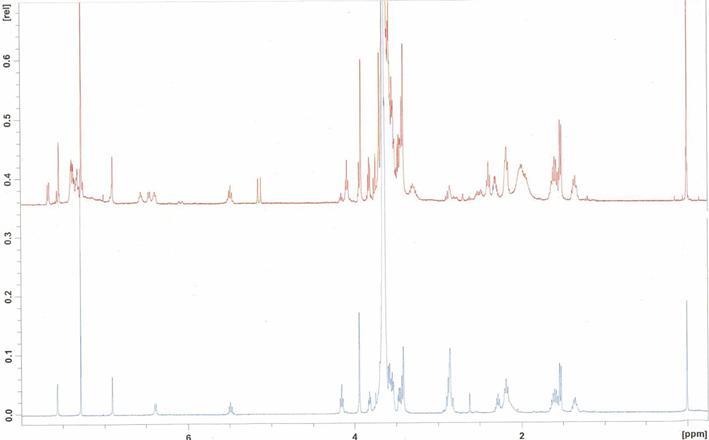


**Figure S3.** ^1^H-NMR spectrum of DBCO-PC-4armPEG. (a) Integrated value in the peak of ^1^H-NMR spectrum for DBCO-PC-4armPEG. (b) Comparison of the spectrum for DBCO-PC-4armPEG (upper spectra) and NHS-PC-4armPEG (bottom spectra).


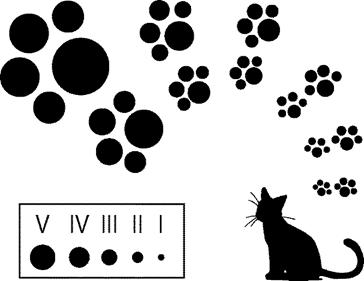


**Figure S4.** The image for micropatterning. The image was drawn by M.T.

**
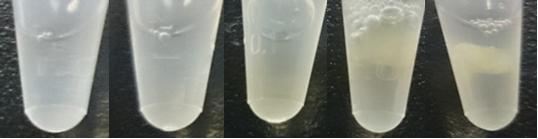
**

**Figure S5.** Pictures showing aqueous solutions of gelatin and DBCO-gelatin at the estimated concentration of 12.5 mg/mL at 37°C. Each picture shows DBCO-gelatin prepared under different preparation condition. Mole ratio of DBCO to primary amino groups in gelatin was 0, 25, 50, 75 and 100 mol% from left to right. The photographs were taken by M.T.
